# Prognostic role of CD74, CD10 and Ki-67 immunohistochemical expression in patients with diffuse malignant peritoneal mesothelioma: a retrospective study

**DOI:** 10.1186/s12885-023-10871-w

**Published:** 2023-05-05

**Authors:** Yufei Liang, Chunying Li, Yingying Liu, Liang Tian, Dongliang Yang

**Affiliations:** 1 Department of Gastroenterology, Cangzhou Central Hospital, Xinhua West Road No.16, Cangzhou, Hebei 061001 China; 2 Department of Pathology, Cangzhou Central Hospital, Xinhua West Road No.16, Cangzhou, Hebei 061001 China; 3 Cangzhou Medical College, Jiuhe West Road No.39, Cangzhou, Hebei 061001 China

**Keywords:** Diffuse malignant peritoneal mesothelioma, Immunohistochemistry, CD74, Ki-67, Prognosis

## Abstract

**Background:**

Diagnosis and treatment of diffuse malignant peritoneal mesothelioma (DMPM) are still challenging. The aim of the present study was to explore the correlation between CD74, CD10, Ki-67 and clinicopathological parameters, and identify independent prognostic factors of DMPM.

**Methods:**

Seventy patients with pathologically proven DMPM were retrospectively reviewed. The expression of CD74, CD10 and Ki-67 in peritoneal tissues was detected by immunohistochemical analysis using standard avidin biotin complex (ABC) immunostaining technique. Kaplan-Meier survival analysis and multivariate Cox regression analyses were performed to assess prognostic factors. The nomogram based on the Cox hazards regression model was established. C-index and calibration curve were performed to evaluate the accuracy of nomogram models.

**Results:**

The median age of DMPM was 62.34 years, and the male-to-female ratio was 1: 1.80. CD74 expression was identified in 52 (74.29%) of 70 specimens, CD10 in 34 (48.57%) specimens, and higher Ki-67 in 33(47.14%) specimens. CD74 was negatively associated with asbestos exposure(*r* = -0.278), Ki-67(*r* = -0.251) and TNM stage(*r* = -0.313). All patients were effectively followed up in the survival analysis. Univariate analysis revealed that PCI, TNM stage, treatment, Ki-67, CD74 and ECOG PS were associated with DMPM prognosis. CD74 (HR = 0.65, 95%Cl:0.46–0.91, *P* = 0.014), Ki-67(HR = 2.09, 95%Cl:1.18–3.73, *P* = 0.012),TNM stage (HR = 1.89, 95%Cl:1.16–3.09, *P* = 0.011), ECOG PS(HR = 2.12, 95%Cl:1.06–4.25, *P* = 0.034), systemic chemotherapy (HR = 0.41, 95%Cl:0.21–0.82, *P* = 0.011) and intraperitoneal chemotherapy (HR = 0.34, 95%Cl:0.16–0.71, *P* = 0.004) were independent predictors by multivariate Cox analysis. The C‑index of the nomogram for predicting overall survival (OS) was 0.81. The OS calibration curve showed good agreement between nomogram-predicted and observed survival.

**Conclusions:**

CD74, Ki-67, TNM stage, ECOG PS and treatment were independent factors affecting prognosis of DMPM. Reasonable chemotherapy treatment might improve the prognosis of patients. The proposed nomogram was a visual tool to effectively predict the OS of DMPM patients.

## Background

Diffuse malignant peritoneal mesothelioma (DMPM) is a rare aggressive tumor originating from the mesothelial cells lining the peritoneal cavity [1]. The lack of specificity of clinical manifestations increases the difficulty of diagnosis, and the tumor has a limited response to standard treatment, the median survival time rarely exceeds 12 months from diagnosis [2]. Cytoreductive surgery (CRS), radiotherapy and chemotherapy may increase the survival. CRS in combination with hyperthermic intraperitoneal chemoperfusion (HIPEC) can prolong survival of selected patients with a 5-year survival rate of 50% [3]. The identification of prognostic factors aids clinicians in the detection of high-risk patients for better management.

The standard for determining the degree of peritoneal carcinomatosis is peritoneal carcinomatosis index (PCI). PCI is an important indicator for predicting survival, higher PCI is significantly associated with poorer OS and progression free survival (PFS) [4].

Cluster of differentiation (CD) proteins play important roles in tumor development by regulating tumor formation, proliferation and metastasis. It has found that the expression of CD74 is linked to some forms of tumors, such as non-small cell lung cancer [5] and pancreatic cancer [6]. CD74 expression in nonhematologic malignancies has been considered to be a prognostic factor, with elevated CD74 as a marker of tumor progression or poor clinical outcome [7]. However, CD74 has been identified as an independent prognostic factor for prolonged OS in patients with malignant pleural mesothelioma [8].

CD10 immuno-expression is reported in several nonhematopoietic neoplasms, such as endometrial stromal sarcoma [9] and renal cell carcinoma [10]. CD10 staining is not only restricted to epithelioid diffuse malignant mesotheliomas (DMMs), but with approximately half of sarcomatoid and biphasic DMMs showing staining [11]. Kadota [12] reported that tumoral CD10 was an independent prognostic factor for patients with malignant pleural mesothelioma.

Ki-67 is a nuclear protein that is detected at every stage of the cell cycle of proliferating cells but is not expressed in G0 phase cells [13]. Ki‑67 is widely used as a prognostic marker in numerous types of cancer, such as gastric cancer [14], ovarian carcinomas [15] and malignant pleural mesothelioma [16]. Nevertheless, the prognostic significance of CD74, CD10 and Ki-67 immunoreactivity in DMPMs has not yet been investigated. In this study, we aim to evaluate the expression of CD74, CD10 and Ki-67 in DMPM patients, and determine independent prognostic factors of DMPM patients.

## Methods

### Patients and tumor tissue samples

The patients with DMPM were screened according to the guidelines for pathologic diagnosis of malignant mesothelioma [17]. Inclusion criteria: (1) DMPM confirmed by pathological and immunohistochemical examination; (2) no other malignant tumors; (3) primary patient with no previous anti-tumor treatment. Exclusion criteria: (1) lack of a clear pathological diagnosis; (2) incomplete data; (3) multiple organ failure and not suitable for treatment. CRS and HIPEC are rarely applied in our region. The statistics referenced for survival with DMPM refers only to the subset of patients not amenable to CRS/HIPEC.

**Fig. 1:**
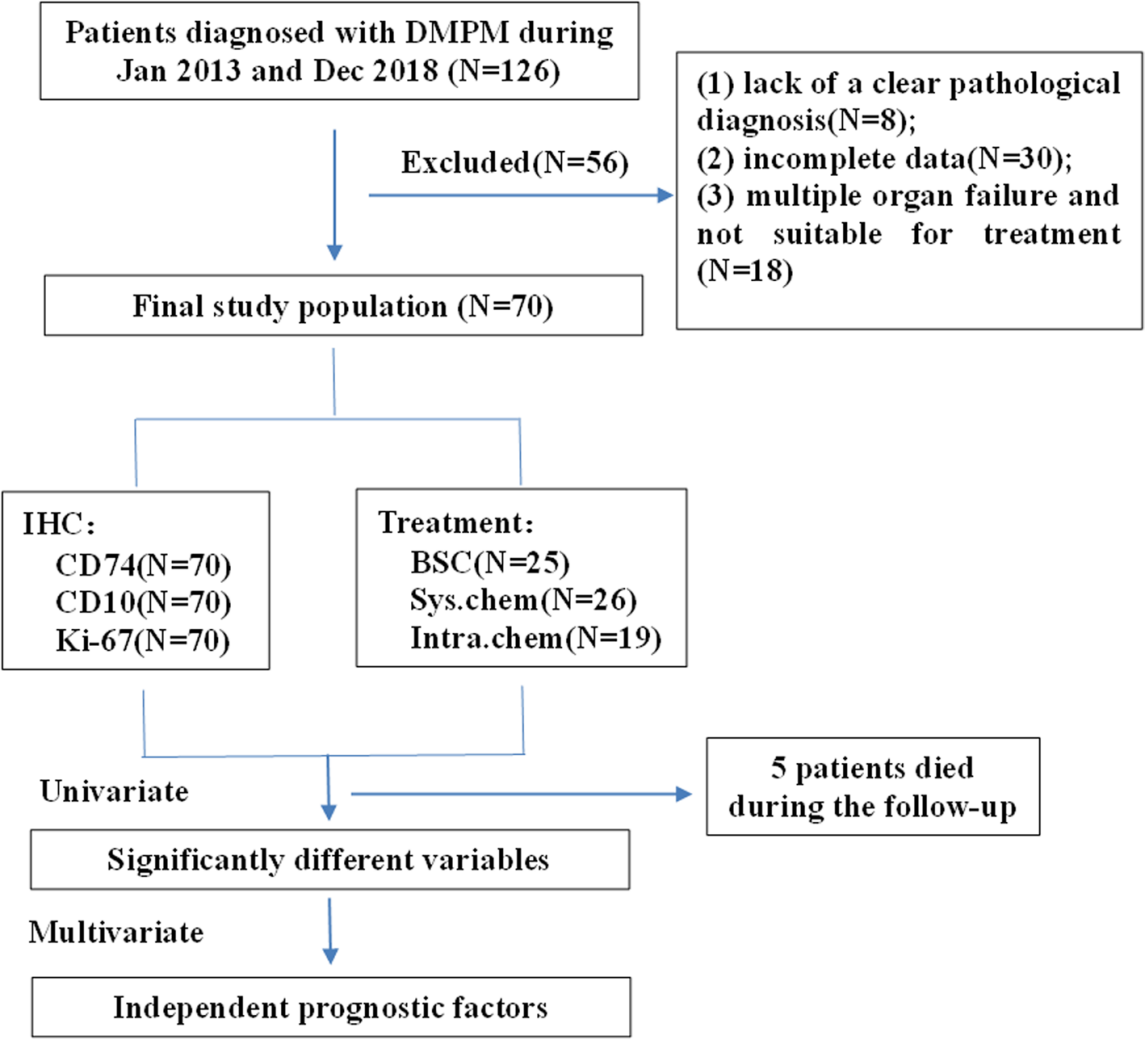
The flow chart of the present study

Of the 126 patients diagnosed with DMPM during January 2013 and December 2018, 56 patients were excluded according to the criteria (Fig. 1), other 70 patients (25 males, 36%; 45 females, 64%) of DMPM met the inclusion criteria. Demographic data, asbestos exposure, histopathological subtype, CD74, CD10, Ki-67, PCI, Eastern Cooperative Oncology Group (ECOG) performance status (PS), and treatment parameters of DMPM were defined as potential prognostic factors and measured at the time of diagnosis. Peritoneal tissue specimens from patients were obtained by laparotomy biopsy, laparoscopic biopsy or ultrasound-guided fine-needle biopsy before patients received any clinical treatment.

The ECOG PS is widely used to quantify the functional status of cancer patients. PS 0 means normal activity, PS 1 means some symptoms, but still near fully ambulatory, PS 2 means less than 50%, and PS 3 means more than 50% of daytime in bed, while PS 4 means completely bedridden.

The stage of DMPM was evaluated by the novel tumor-node-metastasis (TNM) staging system proposed in 2011 [18], which was based on extent of peritoneal disease burden (T), intra-abdominal nodal metastasis (N), and extra-abdominal metastasis (M). Volume evaluation was mainly performed on the basis of computed tomography (CT) images. The extent of peritoneal involvement was scored by the peritoneal cancer index (PCI). PCI rates lesion size from 0 to 3 (no tumor; ≤ 5 mm; > 5–50 mm; > 50 mm) in 13 abdominal-pelvic regions, resulting in a numeric score (PCI 0–39). Yan et al. [18] divided PCI score into four categories: 1–10, 11–20, 21–30, 31–39 corresponding to T stages 1, 2, 3, and 4, respectively. Stage I disease included T1N0M0; stage II included T2–3N0M0 and stage III included T4N0M0 and any N/M positive disease.

### Reagents

The primary antibodies used for immunohistochemistry were shown in Table 1.

**Table 1:** Primary antibodies used for immunohistochemistry

**Target**	**Description**	**Clone No**	**Dilution**	**Manufacturer**
**CD74**	Mouse anti-human monoclonal antibody	SPM523	1:100	ZSGB-BIO, China
**CD10**	Rabbit anti-human monoclonal antibody	SP67	1:50	ZSGB-BIO, China
**Ki-67**	Mouse anti-human monoclonal antibody	7B11	1:200	ZSGB-BIO, China

### Immunohistochemistry (IHC) analyses and evaluation

Immunohistochemical sections were examined by two independent investigators in a blinded manner, any discrepancies were resolved through re-examination and discussion until a consensus was reached.

The standard avidin–biotin-complex peroxidase (ABC) technique was used for immunohistochemical detection. Paraffin-embedded DMPM tissues were cut into 4 μm-thick sections and mounted on glass slides. Sections with the larger number of fixed cells were used for immunohistochemical staining. Each section was deparaffinized, rehydrated and incubated with 0.3% hydrogen peroxide in methanol for 30 min at 25 °C. After washing with PBS, the section was heated in 10 mmol/L citrate buffer (PH = 6) for 10 min. Sections were reacted with antibody against CD74, CD10, and Ki-67. Appropriate controls were used for every case with each staining run. The same protocol was followed for negative controls with omission of the primary antibody. Histologic sections of lymph node, endometrial stroma and breast cancer as mentioned in the antibody datasheets were used as positive controls for CD74, CD10, and Ki-67 staining respectively.

The intensity score (0, no expression; 1, mild expression; 2, intermediate expression; 3, strong expression) and distribution score (0, < 5% immuno‑positive cells; 1, 5–25% immuno‑positive cells; 2, 26–50% immuno‑positive cells; 3, 51–100% immuno‑positive cells) for immunostaining were summed into a total score (range 0–6). The positivity intensity was graded according to the total score (0, score 0–1; 1+, score 2–3; 2+, score 4–5; 3+, score 6). Grade 0 was defined as negative, and grades 1+, 2+ and 3+ were defined as positive. According to the positive cell rate of Ki-67 in per 500 tumor cells, we divided Ki-67 into low Ki-67 group (≤ 15%) and high Ki-67 groups (> 15%).

### Follow up and end point

After the first treatment, we conducted regular follow-up evaluation for all patients every 3 months through telephone interviews or short massage platform. The last follow-up time and vital status were recorded. OS was defined as the duration from the date of diagnosis to the end point (in months). The end point was defined as patient’s death or December 31, 2019 in this study. Patients who were still alive or lost at the last visit were censored at the date of last follow-up.

### Statistical analysis

Descriptive statistics for demographic and clinicopathological data were performed. Categorical variables were presented as numbers (percentages), continuous variables were presented as mean ± standard deviation. Correlations between parameters were tested by Spearman rank correlations (Spearman’s rho). Kaplan-Meier analysis was used to calculate the overall cumulative probability of survival, and the log-rank test was used to assess differences in survival. Univariate analysis was performed to assess the association between prognostic parameters and survival. Cox Proportional hazards regression model (stepwise backward method) was used for the multivariate analysis to evaluate the prognostic value of related factors. The clinically significant variables calculated from the Cox proportional hazards model were integrated into a nomogram. The nomogram was formulated to predict the prognosis of DMPM, forest plot of hazard ratio was used to illustrate the results. The predictive accuracy of the model was estimated using the concordance index (C‑index). The calibration curve was used to evaluate the consistency between the predicted survival rate and the actual survival rate. Two tailed *P* values less than 0.05 were considered statistically significant. Statistical analyses were performed using SPSS 22.0 (IBM, Armonk, NY) and R version 4.1.3 software (http://www.r-project.org/). Extension packages, including “survival”, “nomogramEx”, “rms” and “survminer” were used.

## Results

### Patients

Clinical and pathologic features of patients were summarized in Table 2. The ratio of male to female was 1:1.80. There was no significant difference in age between male and female patients (61.68 years vs 62.71 years, respectively). 62 patients had confirmed asbestos exposure. 26 patients received systemic chemotherapy with pemetrexed plus cisplatin, 19 patients received intraperitoneal chemotherapy with cisplatin, other 25 patients only received best supportive care (BSC) treatment. 5 patients were still alive at the time of the final analysis.

**Table 2:** Demographic and clinical pathological features of the malignant peritoneal mesothelioma patients

**Factors**	**(Mean ± SD) or (n, %)**
**Age at diagnosis (AAD)** (mean ± SD, years)	62.34 ± 9.96
**Age group** (n, %)	
≤ 60 years	31 (44.29)
> 60 years	39 (55.71)
**Gender** (n, %)	
Male	25 (35.71)
Female	45 (64.29)
**Asbestos exposure** (n, %)	
Yes	62 (88.57)
No	8 (11.43)
**Performance status** (ECOG) (n, %)	
< 2	29 (41.43)
≥ 2	41 (58.57)
**Histopathological type** (n, %)	
Epithelial	37 (52.86)
Non-epithelial	33 (47.14)
**Platelet** (n, %)	
< 300 × 10^9^/L	23 (32.86)
≥ 300 × 10^9^/L	47 (67.14)
**Peritoneal carcinomatosis index (PCI)** (n, %)	
≤ 25	31 (44.29)
25	39 (55.71)
**TNM stage** (n, %)	
I	20 (28.57)
II	39 (55.71)
III	11 (15.71)
**Treatment** (n, %)	
BSC	25 (35.71)
Systemic chemotherapy	26 (37.14)
Intraperitoneal chemotherapy	19 (27.14)
**Status** (n, %)	
Alive	5 (7.14)
Dead	65 (92.86)

### Correlations between CD74 and CD10 expression and clinicopathological parameters

**Fig. 2:**
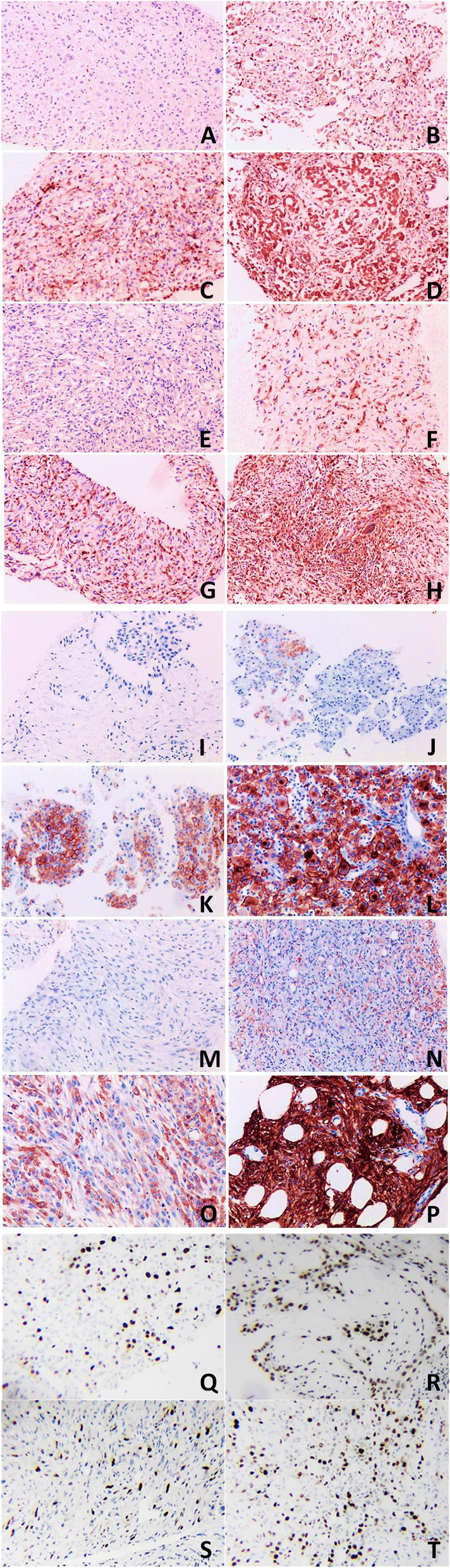
Immunohistochemical expression of CD74, CD10 and Ki-67 in epithelial and non-epithelial DMPM (original magnification × 200). CD74 and CD10 showed the cytoplasm and(or) cell membrane staining. Ki-67 showed nuclei staining of cell. Expression of CD74 in epithelioid DMPM: **A** Negative; **B** 1+; **C** 2+; **D** 3+. Expression of CD74 in Non-epithelioid DMPM: **E** Negative; **F** 1+; **G** 2+; **H** 3+. Expression of CD10 in epithelioid DMPM: **I** Negative; **J** 1+; **K** 2+; **L** 3+. Expression of CD10 in Non-epithelioid DMPM: **M** Negative; **N** 1+; **O** 2+; **P** 3+. Expression of Ki-67 in epithelioid DMPM: **Q** lower expression; **R** higher expression. Expression of Ki-67 in Non-epithelioid DMPM: **S** lower expression; **T** higher expression

In tissue samples, both CD74 and CD10 were mainly expressed in the cytoplasm/membrane and were heterogeneous within positive staining tumors, while Ki-67 staining was only nuclear. CD74 was positive in peritoneal tissue of 52 DMPM patients (1+, 26 cases; 2+, 20 cases; 3+, 6 cases), CD10 was positive in peritoneal tissue of 34 DMPM patients (1+,18 cases; 2+, 10 cases; 3+, 6 cases), and higher Ki-67(> 0.15) was in peritoneal tissue of 33 DMPM patients (Fig. 2). Spearman’s rho analysis revealed that CD74 was negatively correlated with TNM stage (*P* = 0.008, *r* = -0.313), asbestos exposure (*P* = 0.020, *r* = -0.278) and Ki-67 (*P* = 0.036, *r* = -0.251) (Table 3).

**Table 3:** Correlation analysis of CD74, CD10 and clinicopathologic parameters in patients with malignant peritoneal mesothelioma

	**N**	**CD74**				***P***	**CD10**				***P***
		**0**	**1+**	**2+**	**3+**		**0**	**1+**	**2+**	**3+**	
**Age**						0.195					1.000
≤ 60 year	31	6	11	11	3		16	8	4	3	
> 60 year	39	12	15	9	3		20	10	6	3	
**Gender**						0.593					0.243
Male	25	9	6	8	2		16	4	2	3	
Female	45	9	20	12	4		20	14	8	3	
**Performance status** (ECOG)						0.392					0.817
< 2	29	8	7	11	3		14	8	6	1	
≥ 2	41	10	19	9	3		22	10	4	5	
**Histopathological type**						0.101					0.477
Epithelioid	37	12	14	9	2		20	10	5	2	
Non-epithelioid	33	6	12	11	4		16	8	5	4	
**PCI**						0.314					0.103
≤ 25	31	10	9	9	3		18	8	2	3	
25	39	8	17	11	3		18	10	8	3	
**TNM Stage**						**0.008**^**△**^					0.198
I	20	2	6	8	4		12	6	2	0	
II	39	13	14	10	2		19	9	7	4	
III	11	3	6	2	0		5	3	1	2	
**PLT**						0.086					0.292
< 300 × 10^9^/L	23	7	11	5	0		14	4	5	0	
≥ 300 × 10^9^/L	47	11	15	15	6		22	14	5	6	
**Asbestos exposure**						0.020*					0.100
Yes	62	18	24	14	6		31	15	10	6	
No	8	0	2	6	0		5	3	0	0	
**Ki67**						**0.036**^**#**^					0.585
≤ 0.15	37	9	9	13	6		19	12	4	2	
> 0.15	33	9	17	7	0		17	6	6	4	

^△^*r* = -0.313

^*^r = -0.278

^#^*r* = -0.251

### Univariate analysis

**Fig. 3:**
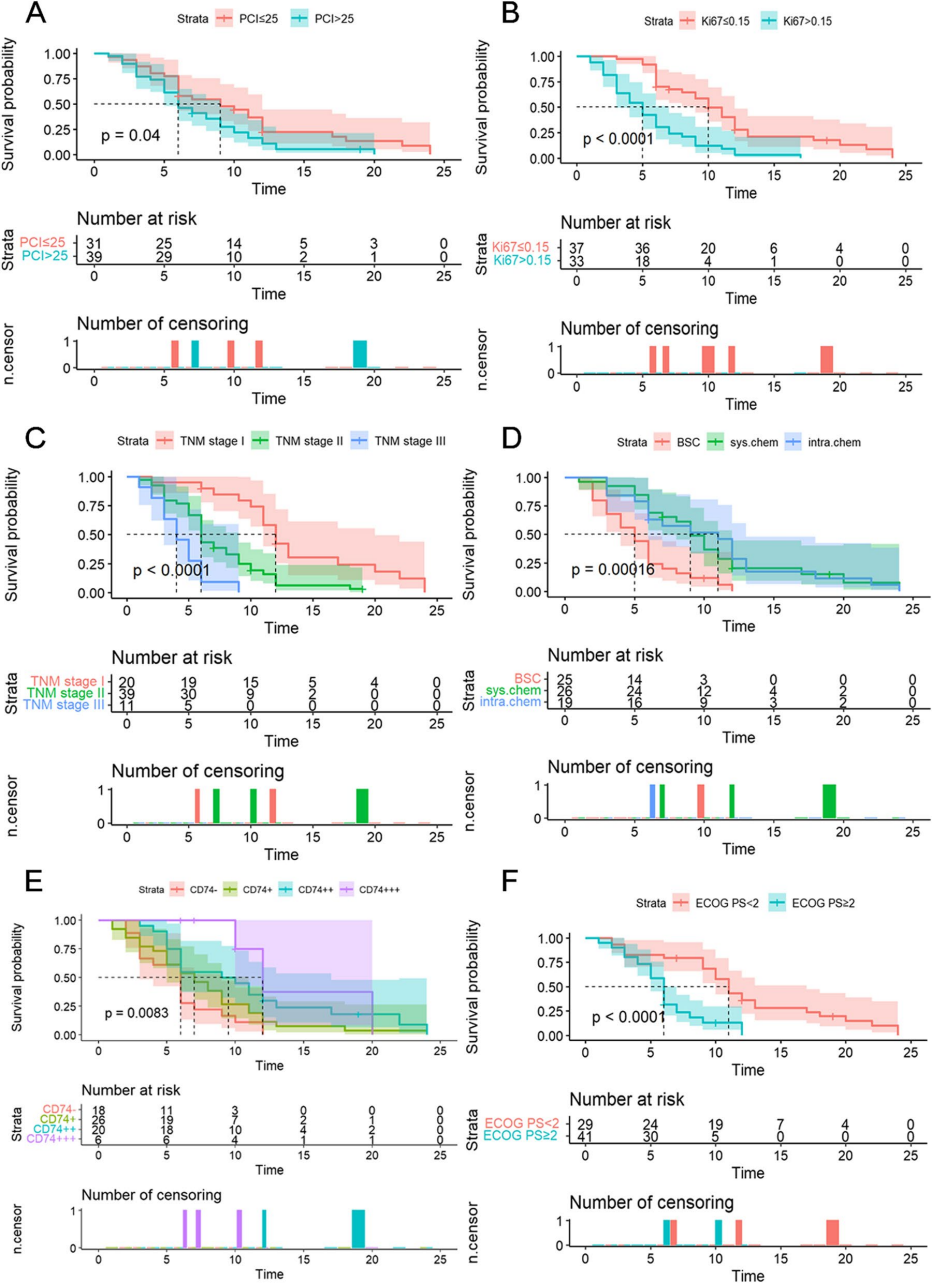
Kaplan-Meier survival curves for five significant predictors. The curves showed the overall survival rates in DMPM patients. Dashed lines represented median survival time. **A** PCI, **B** Ki-67, **C** TNM stage, **D** treatment, **E** CD74, **F** ECOG PS. Sys.chem: systemic chemotherapy; intra.chem: interperitoneal chemotherapy

All patients were effectively followed up in the survival analysis. The median survival time was 7.00 ± 0.73 months (range, 1–24). To assess OS, 12 potential prognostic parameters (age, gender, histopathological type, ECOG PS, asbestos exposure, PCI, TNM stage, CD74, CD10, PLT, Ki-67, treatment) were included in the univariate analysis. As shown in Fig. 3 and Table 4, ECOG PS, PCI, TNM stage, CD74, Ki-67, systemic chemotherapy and intraperitoneal chemotherapy were found to be significantly associated with DMPM prognosis. Number at risk tables and censored graphs were displayed below the Kaplan-Meier curves. DMPM patients with lower PCI, lower Ki-67 and earlier TNM stage exhibited longer survival time, while DMPM patients with lower CD74, higher ECOG PS and without chemotherapy treatment expression had poorer prognosis.

**Table 4:** Univariate and multivariate analysis of parameters in patients with malignant peritoneal mesothelioma

**Variable**	**Univariate analysis**			**Multivariate analysis**		
	**O/N**	**Survival**^**a**^	***P***	**HR**	**95%Cl**	***P***
**Age**			0.394			
≤ 60 year	27/31	9.37				
> 60 year	38/39	8.19				
**Gender**			0.256			
Male	21/25	7.53				
Female	44/45	9.21				
**Performance status** (ECOG)			**0.000**	2.12	1.06–4.25	**0.034**
< 2	26/29	12.11				
≥ 2	39/41	6.11				
**Histopathological type**			0.958			
Epithelioid	35/37	8.53				
Non-epithelioid	30/33	8.78				
**Asbestos exposure**			0.957			
Yes	58/62	8.68				
No	7/8	7.75				
**PCI**			**0.040**	1.48	0.84–2.59	0. 172
≤ 25	28/31	10.32				
25	37/39	7.38				
**TNM stage**			**0.000**	1.89	1.16–3.09	**0.011**
Stage I	18/20	13.48				
Stage II	36/39	7.29				
Stage III	11/11	4.36				
**CD74**			**0.008**	0.65	0.46–0.91	**0.014**
–	18/18	5.89				
+	26/26	7.73				
++	18/20	10.89				
+++	3/6	14.50				
**CD10**			0.090			
–	32/36	9.41				
+	18/18	9.33				
++	9/10	6.50				
+++	6/6	5.50				
**PLT**			0.309			
< 300 × 10^9^/L	22/23	7.83				
≥ 300 × 10^9^/L	43/47	9.11				
**Ki67**			**0.000**	2.09	1.18–3.73	**0.012**
≤ 0.15	32/37	11.50				
> 0.15	33/33	5.61				
**Treatment**			**0.000**			**0.013**
BSC	24/25	5.34		1.00		
Systemic chemotherapy	23/26	10.51	0.000*	0.41	0.21–0.82	**0.011**
Intraperitoneal chemotherapy	18/19	10.47	**0.001**^**#**^	0.34	0.16–0.71	**0.004**

*O* Observed death number, *N* Total patient number, *HR* Hazard ratio

^*^Systemic chemotherapy vs. BSC

^#^Intraperitoneal chemotherapy vs. BSC

^a^Mean survival (months)

### Multivariate analysis

**Fig. 4:**
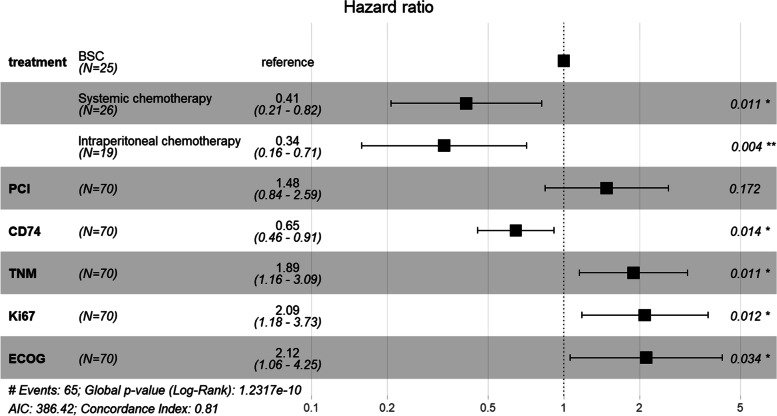
The hazard ratio of independent risk factors (Treatment, PCI, CD74, TNM, Ki-67, ECOG) in Cox proportional hazards model. Forest plot of subgroup showed that treatment, CD74, TNM, Ki-67 and ECOG were independent factors for prognosis. C-index = 0.81

**Fig. 5:**
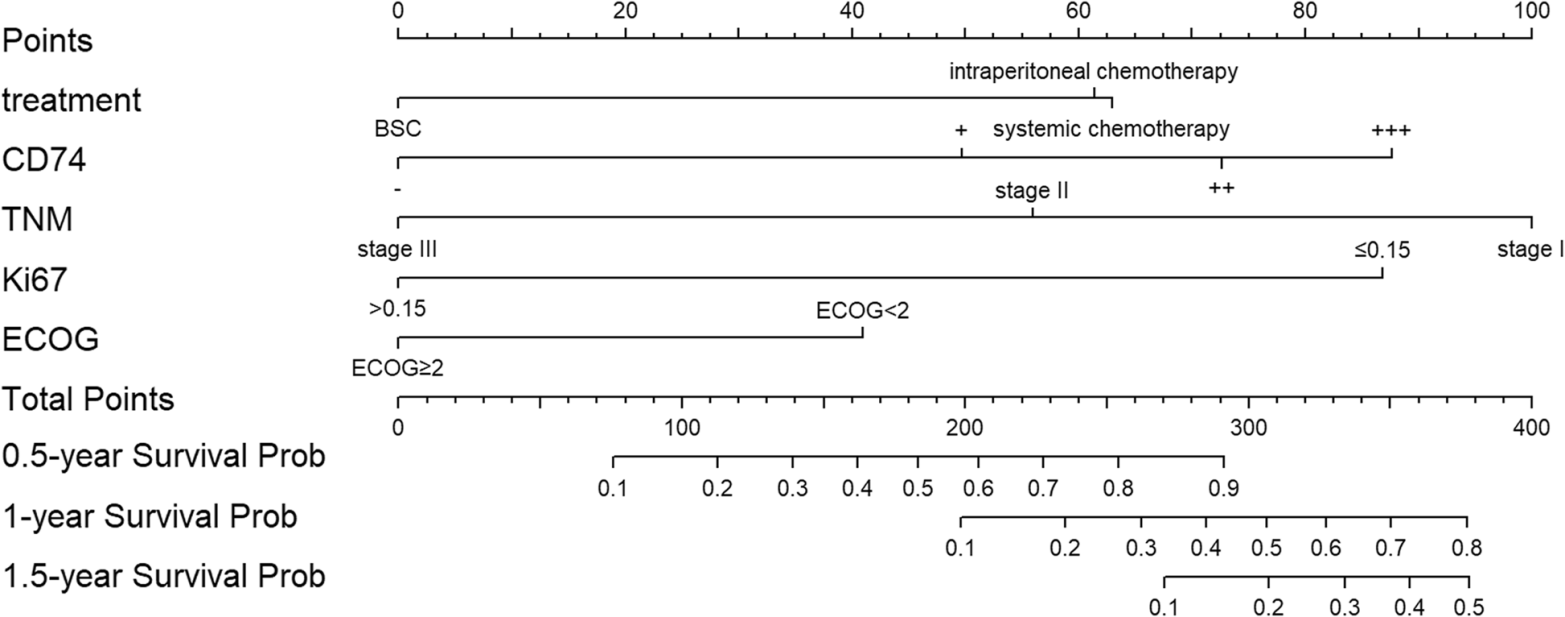
Nomogram figure used to predict patient survival probability of 0.5-, 1- and 1.5- year for DMPM patients. The nomogram was used by adding up the points identified on the points scale for each variable. The total points projected on the bottom scales indicate the year survival probability

**Fig. 6:**
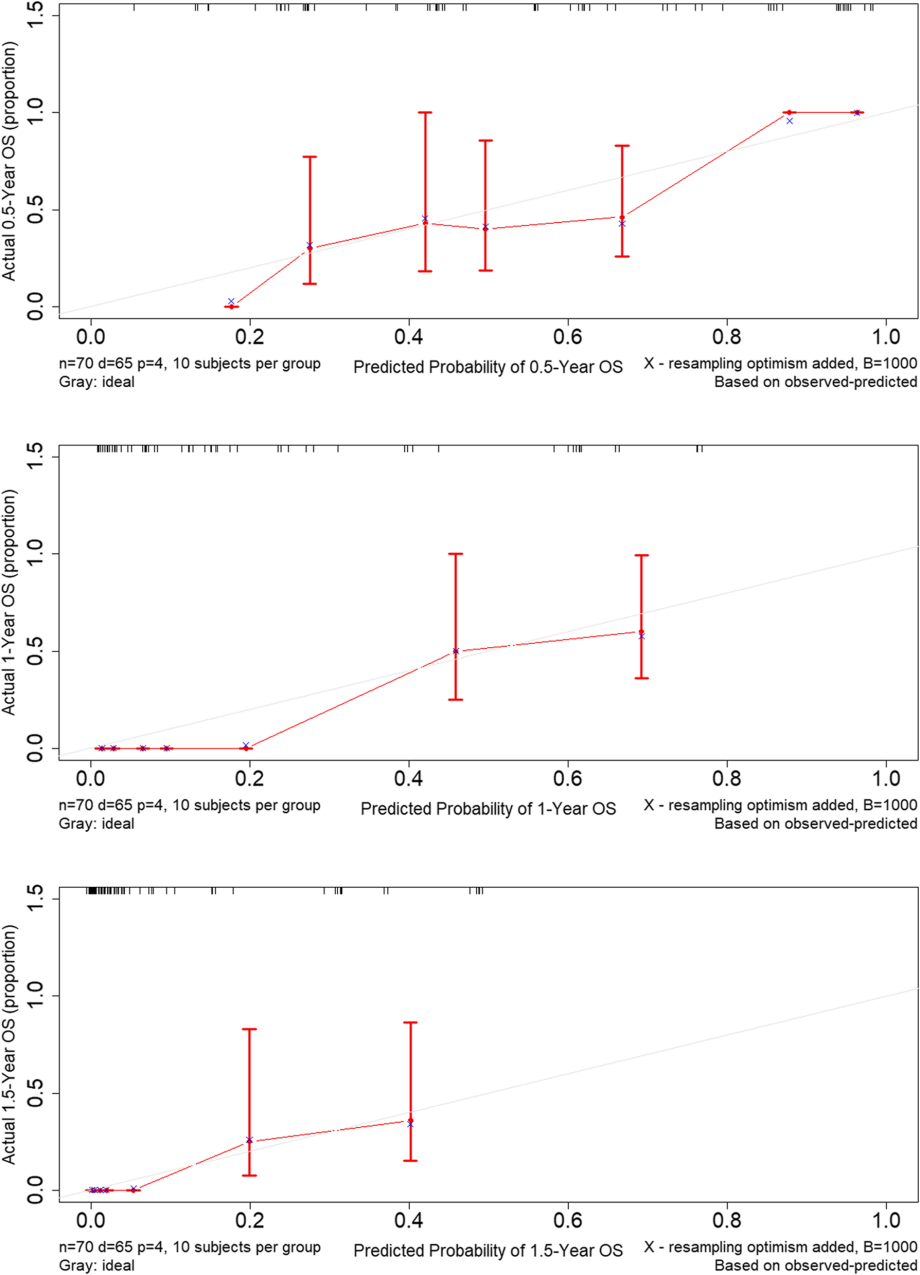
Calibration curves for predicting 0.5-, 1- and 1.5-year overall survival for DMPM patients. X-axis represented the nomogram-predicted survival; Y-axis represented the actual OS using the Kaplan-Meier method. Light gray diagonal represented an ideal nomogram. Red vertical solid line was current nomogram performance with 95% confidence intervals

Variables with *P* values < 0.05 in the univariate analysis were included in the model for the multivariate analysis to identify independent influencing factors. All 70 patients were followed up successfully with complete information. At the time of last follow-up, five patients were still alive. Multivariate analysis showed that CD74(HR = 0.65, 95%CI: 0.46–0.91, *P* = 0.014), TNM stage (HR = 1.89, 95%CI: 1.16–3.09,* P* = 0.011), Ki-67(HR = 2.09, 95%CI: 1.18–3.73,* P* = 0.012), ECOG PS(HR = 2.12, 95%CI: 1.06–4.25,* P* = 0.034) and treatment protocols, including systemic chemotherapy(HR = 0.41, 95%CI: 0.21–0.82,* P* = 0.011) and intraperitoneal chemotherapy (HR = 0.34, 95%CI: 0.16–0.71,* P* = 0.004) were independent prognostic factors of DMPM (Table 4; Fig. 4). We constructed a nomogram for OS based on independent prognostic factors obtained from multivariate Cox regression model analysis (Fig. 5), The nomograms showed that TNM stage contributed the most to predicting OS in patients with DMPM, followed by Ki-67, CD74, treatment and ECOG PS. The weighted score for each significant variable ranged from 0 to 100 and the corresponding risk rate ranged from 0.1 to 0.9. By adding up the total score from all the variables and locating it to the total point scale, the probabilities of the outcomes could be determined. which implies the prognosis for survival probabilities at 0.5-, 1-, and 1.5-year for patients with DMPM. The lower the total point, the poorer the prognosis. The predictive ability of the model was assessed by calculating the C-index, which was 0.81 (95%CI:0.76–0.85). Bootstrap method was used for internal verification of nomogram diagram with the number of self-sampling B = 1000, and 10 subjects per group. The performance of the nomogram was graphically evaluated using a calibration curve, which displayed consistency between the nomogram predicted survival and the observed survival rate for 0.5-, 1-, and 1.5-year OS (Fig. 6). The predicted line overlapped well with the reference line, demonstrating the good performance of the nomogram.

## Discussion

Malignant Peritoneal Mesothelioma (MPM) is a tumor originating from peritoneal epithelium and mesothelium, it is easily missed or misdiagnosed because of low incidence. The incidence rate of MPM in the United States was 1–2/100 million [19], but was 4.5/100 million in Eastern China in 2018 [2]. MPM is divided into the diffuse type and localized type, and most patients show diffuse peritoneal involvement. In our study, the high asbestos exposure rate and the frequent incidence of disease in women could be attributed to the fact that in the 1970s, most of the asbestos processing factories in Eastern China employed female workers, the occupational exposure rate of female workers was higher than that of male workers. Although some patients with early-stage epithelioid disease may have a median survival of up to 2 years [20], most patients die within 1 year of diagnosis [2]. Therefore, It is necessary to identify prognostic factors of DMPM. Numerous studies have suggested several prognostic factors for peritoneal mesothelioma, including sex [21], histologic tumor grade [3], blood neutrophil‑to‑lymphocyte ratio [22] and platelet-to-lymphocyte ratio [23], among others. Our study investigated the prognostic effects of immunohistochemical and clinical indicators in DMPM patients.

Epidemiological studies have confirmed that the occurrence and development of tumors are inseparable from inflammation [24]. Continuous stimulation of the peritoneum by asbestos fibers can cause inflammatory reactions. CD74 is a type II transmembrane glycoprotein associated with the major histocompatibility complex (MHC) class II α and β chains, it is expressed in the cytoplasm and cell membrane, and participates in several key processes of the immune system, including antigen presentation, B-lymphocyte differentiation and inflammatory signaling [25]. As a receptor of inflammatory cytokines, CD74 combines with proinflammatory factors, initiates a signal cascade to promote the formation of tumor lesions [26]. In recent years, it has been noted that CD74 is expressed in some solid tumors, and high expression level of CD74 correlates with poor prognosis [27–29]. Nevertheless, Otterstrom reported that high expression of CD74 was an independent prognostic factor for prolonged OS in mesothelioma pleural patients (low CD74: 8.2 months; medium CD74: 14.0 months; high CD74: 14.7 months; *P* < 0.001) [8]. Moreover, the prognosis of hepatocellular carcinoma (HCC) patients with positive CD74 expression was better than that with negative CD74 expression, and it could be a biomarker of the prognosis in HCC [30]. The contribution of CD74 to cancerogenesis seemed to vary with the type of cancer and stage of the disease [8]. Our results demonstrated that CD74 was an independent prognostic factor. Lack of CD74 expression might indicate a poor prognosis. In contrast, over expression of CD74 predicted a better prognosis, consistent with previous literature [8]. This indicator may prompt clinicians to provide theoretical guidance for improving the prognosis of patients.

CD10 is a zinc dependent metalloproteinase expressed on cell surface, it can degrade bioactive peptides in the extracellular matrix. CD10 staining was observed in 54% of diffuse malignant mesotheliomas, slightly more than half of which(55%) showed high immuno-expression [11]. In the present study, approximately half of DMPM patients (34/70) were immunoreactive for CD10. The CD10-positive expression tended to be weak both of epithelial and non-epithelial DMPM, and CD10 expression was not associated with prognosis in either univariate or multivariate analyses, inconsistent with the previous literature [12]. The reason may be related to the small number of case samples and the type of histology. Therefore, larger sample size should be increased in the future to reduce the error.

PCI is one of the indicators to evaluate the extent of peritoneal mesothelioma lesions. Silja reported that PCI had a significant association with survival and significant prognostic value in patients with MPM [31]. PCI summary describes the size and distribution of tumor planting nodules in the 13 regions of the abdominal cavity, and combines the lesion size in each zone to record the total score (range, 0–39), which quantifies the severity of the peritoneal tumor [32]. This method can be more accurate to measure cancers invading peritoneum. CT and laparoscopy seem to be effective tools for assessment of peritoneal carcinomatosis using the PCI score, which has no statistically significant differences regarding total PCI score compared to surgery [33]. So, PCI was recorded by CT in the present study. The involvement of different areas in the PCI system has a significant impact on the prognosis and survival [34]. In present study, univariate analysis demonstrated that PCI and TNM stage could influence prognosis, while TNM stage was an independent prognostic variable by multivariate analysis, which was in line with previous literature [18]. The author speculates that PCI may be an intermediary variable affecting prognosis by influencing TNM stage, suggesting that TNM staging has an important impact on the prognosis of peritoneal mesothelioma.

ECOG PS is an important factor determining prognosis in a number of malignant conditions, including mesothelioma patients [35]. Patients who scored between 2–4 on the ECOG scale had shorter survival times when compared to those with score between 0–1 [36]. Our study revealed survival was obviously shorter in those who had an ECOG score greater than 2, which was an independent predictor of prognosis.

The expression level of Ki-67 represents the proliferation status of tissues and can be used to determine the malignancy of tumors. DMPM Ki-67 has been demonstrated to be an important prognostic marker [37]. Our study confirmed previous finding and suggested that patients with Ki-67 > 15% were unlikely to benefit from the treatment.

In general, epithelial DMPM has a better prognosis than other types [38]. The inconsistencies in this study may be related to the selected cases. Most epithelial cases were treated only after severe conditions had developed, resulting in a short survival period.

Currently, there is no standard therapy for DMPM, the optimal treatment of DMPM remains controversial. CRS and HIPEC are internationally recommended for the treatment of DMPM, they can effectively improve survival [3]. Pemetrexed combined with cisplatin are approved as first-line therapy for HIPEC in patients with DMPM [22]. In HIPEC, chemotherapy drugs are in direct contact with tumor tissues, with high local concentrations. Due to the existence of blood-peritoneal barrier, side effects are less compared with intravenous chemotherapy. For patients who do not undergo surgery, clinicians usually provide supportive treatment or chemotherapy. The first‑line clinical systemic therapy is pemetrexed combined with cisplatin or carboplatin [39].This study showed that chemotherapy treatment was an independent risk factor for the prognosis of DMPM patients. Relative to BSC, patients could benefit from systemic or intraperitoneal chemotherapeutic interventions, suggesting that the risk of death after chemotherapy was reduced. Sugarbaker found that long-term regional chemotherapy was associated with improved survival in patients with DMPM [40]. Therefore, patients who do not receive surgery could be guided and advocated for chemotherapy in order to prolong survival time.

It is always hard to construct a model with universal applicability and high accuracy, and establishment of a survival prediction nomogram of rare tumors based on a single mechanism may be another option [41]. In present study, we constructed a nomogram which demonstrated high accuracy for OS protection (C‑index, 0.81). Nomogram may assist physicians in selecting appropriate treatment for DMPM patients with regard to the probability of a survival benefit, and has a prognostic potential to predict survival accordingly [42]. The calibration curves represent a good fit between observed proportion and predicted probability. The results of Cox survival analysis are visualized by forest map, HR values of independent prognostic risk factors are also displayed.

Our study has several limitations that may affect prognosis. First, it is a retrospective and single-center study with a small clinical database, institutional heterogeneity and number of patients may affect the results. Second, the lack of CRS/HIPEC information in the present study may affect the prognosis of the DMPM, and these important factors should be considered in future studies. Finally, the factors affecting the prognosis of DMPM are varied and complicated, the indexes we choose are relatively limited and biased to some extent, which may affect the prognosis of the disease. Therefore, comprehensive research on large samples, multi-center, multi-indicators and multi discipline may improve the evaluative accuracy.

## Conclusions

In conclusion, we determined that CD74, Ki-67, TNM stage, ECOG PS and tumor‑directed treatment were associated with DMPM prognosis. Reasonable chemotherapy treatment may improve the prognosis of DMPM patients. The nomogram is a visual tool to effectively predict OS in DMPM patients, but needs to be tested in prospective clinical trials.

## Data Availability

The datasets generated during and/or analyzed during the current study are available from the corresponding author on reasonable request.

## References

[1] Liang YF, Zheng GQ, Chen YF (2016). CT differentiation of diffuse malignant peritoneal mesothelioma and peritoneal carcinomatosis. J Gastroenterol Hepatol.

[2] Hui S, Guo-Qi Z, Xiao-Zhong G (2018). IMP3 as a prognostic biomarker in patients with malignant peritoneal mesothelioma. Hum Pathol.

[3] Alexander HR, Bartlett DL, Pingpank JF (2013). Treatment factors associated with long-term survival after cytoreductive surgery and regional chemotherapy for patients with malignant peritoneal mesothelioma. Surgery.

[4] Ng JL, Ong WS, Chia CS (2016). Prognostic relevance of the peritoneal surface disease severity score compared to the peritoneal cancer index for colorectal peritoneal carcinomatosis. Int J Surg Oncol.

[5] McClelland M, Zhao L, Carskadon S (2009). Expression of CD74, the receptor for macrophage migration inhibitory factor, in non-small cell lung cancer. Am J Pathol.

[6] Zhang JF, Hua R, Liu DJ (2014). Effect of CD74 on the prognosis of patients with resectable pancreatic cancer. Hepatobiliary Pancreat Dis Int.

[7] Stein R, Mattes MJ, Cardillo TM (2007). CD74: a new candidate target for the immunotherapy of B-cell neoplasms. Clin Cancer Res.

[8] Otterstrom C, Soltermann A, Opitz I (2014). CD74: a new prognostic factor for patients with malignant pleural mesothelioma. Br J Cancer.

[9] McCluggage WG, Sumathi VP, Maxwell P (2001). CD10 is a sensitive and diagnostically useful immunohistochemical marker of normal endometrial stroma and of endometrial stromal neoplasms. Histopathology.

[10] Langner C, Ratschek M, Rehak P (2004). CD10 is a diagnostic and prognostic marker in renal malignancies. Histopathology.

[11] Butnor KJ, Nicholson AG, Allred DC (2006). Expression of renal cell carcinoma-associated markers erythropoietin, CD10, and renal cell carcinoma marker in diffuse malignant mesothelioma and metastatic renal cell carcinoma. Arch Pathol Lab Med.

[12] Kadota K, Villena-Vargas J, Nitadori J (2015). Tumoral CD10 expression correlates with aggressive histology and prognosis in patients with malignant pleural mesothelioma. Ann Surg Oncol.

[13] Yerushalmi R, Woods R, Ravdin PM (2010). Ki-67 in breast cancer: prognostic and predictive potential. Lancet Oncol.

[14] Lazăr D, Tăban S, Sporea I (2010). Ki-67 expression in gastric cancer. Results from a prospective study with long term follow-up. Rom J Morphol Embryol.

[15] Aune G, Stunes AK, Tingulstad S (2011). The proliferation markers Ki-67/MIB-1, phosphohistone H3, and survivin may contribute in the identification of aggressive ovarian carcinomas. Int J Clin Exp Pathol.

[16] Ghanim B, Klikovitsl T, Hoda MA (2015). Ki-67 index is an independent prognostic factor in epithelioid but not in non-epithelioid malignant pleural mesothelioma: a multicenter study. Br J Cancer.

[17] Husain AN, Colby T, Ordonez N (2013). Guidelines for pathologic diagnosis of malignant mesothelioma: 2012 update of the consensus statement from the International Mesothelioma Interest Group. Arch Pathol Lab Med.

[18] Yan TD, Deraco M, Elias D (2011). A novel tumor-node-metastasis (TNM) staging system of diffuse malignant peritoneal mesothelioma using outcome analysis of a multi-institutional database. Cancer.

[19] Chua TC, Yan TD, Morris DL (2009). Surgical biology for the clinician: peritoneal mesothelioma: current understanding and management. Can J Surg.

[20] Weder W, Opitz I, Stahel R (2009). Multimodality strategies in malignant pleural mesothelioma. Semin Thorac Cardiovasc Surg.

[21] Cao C, Yan TD, Deraco M (2012). Importance of gender in diffuse malignant peritoneal mesothelioma. Ann Oncol.

[22] Yin WJ, Zheng GQ, Yang KN (2018). Analysis of prognostic factors of patients with malignant peritoneal mesothelioma. World J Surg Oncol.

[23] Liang Y, Zheng G, Yin W (2019). Significance of EGFR and PTEN expression and PLR and NLR for predicting the prognosis of epithelioid malignant peritoneal mesothelioma. Gastroenterol Res Pract.

[24] Greten FR, Grivennikov SI (2019). Inflammation and cancer: triggers, mechanisms, and consequences. Immunity.

[25] Borghese F, Clanchy FIL (2011). CD74: an emerging opportunity as a therapeutic target in cancer and autoimmune disease. Expert Opin Ther Targets.

[26] Greenwood C, Metodieva G, Al-Janabi K (2012). Stat1 and CD74 overexpression is co-dependent and linked to increased invasion and lymph node metastasis in triple-negative breast cancer. J Proteomics.

[27] Shakib PA, Ensani F, Abdirad A (2015). CD44 and CD74: the promising candidates for molecular targeted therapy in oral squamous cell carcinoma. Dent Res J (Isfahan).

[28] Wang ZQ, Milne K, Webb JR (2017). CD74 and intratumoral immune response in breast cancer. Oncotarget.

[29] Nagata S, Jin YF, Yoshizato K (2009). CD74 is a novel prognostic factor for patients with pancreatic cancer receiving multimodal therapy. Ann Surg Oncol.

[30] Fu XT, Dai Z, Zhao YM (2012). Cluster of differentiation 74 Plays a role in prognosis of the patients with hepatocellular carcinoma after curative resection. Chin J Lab Med.

[31] Salo SAS, Lantto E, Robinson E (2020). Prognostic role of radiological peritoneal cancer index in malignant peritoneal mesothelioma: national cohort study. Sci Rep.

[32] Pasqual EM, Bertozzi S, Bacchetti S (2014). Preoperative assessment of peritoneal carcinomatosis in patients undergoing hyperthermic intraperitoneal chemotherapy following cytoreductive surgery. Anticancer Res.

[33] Ahmed SA, Abou-Taleb H, Yehia A (2019). The accuracy of multidetector computed tomography and laparoscopy in the prediction of peritoneal carcinomatosis index score in primary ovarian cancer. Acad Radiol.

[34] Spiliotis J, Halkia EE, Kalantzi N (2015). Mapping the location of peritoneal metastases using the peritoneal cancer index and the correlation with overall survival: a retrospective study. J Buon.

[35] Musk AW, Olsen N, Alfonso H (2011). Predicting survival in malignant mesothelioma. Eur Respir J.

[36] Sebbag G, Yan H, Shmookler BM (2000). Results of treatment of 33 patients with peritoneal mesothelioma. Br J Surg.

[37] Pezzuto F, Serio G, Fortarezza F (2020). Prognostic value of Ki67 percentage, WT-1 expression and p16/CDKN2A deletion in diffuse malignant peritoneal mesothelioma: a single-centre cohort study. Diagnostics (Basel).

[38] Tischoff I, Neid M, Neumann V (2011). Pathohistological diagnosis and differential diagnosis. Recent Results Cancer Res.

[39] Levý M, Boublíková L, Büchler T (2019). Treatment of malignant peritoneal mesothelioma. Klin Onkol.

[40] Sugarbaker PH, Chang D (2017). Long-term regional chemotherapy for patients with epithelial malignant peritoneal mesothelioma results in improved survival. Eur J Surg Oncol.

[41] Bai Y, Liu ZS, Xiong JP (2018). Nomogram to predict overall survival after gallbladder cancer resection in China. World J Gastroenterol.

[42] Balachandran VP, Gonen M, Smith JJ (2015). Nomograms in oncology: more than meets the eye. Lancet Oncol.

